# Conformation dependent monoclonal antibodies distinguish different replicating strains or conformers of prefibrillar Aβ oligomers

**DOI:** 10.1186/1750-1326-5-57

**Published:** 2010-12-13

**Authors:** Rakez Kayed, Isabel Canto, Leonid Breydo, Suhail Rasool, Tamas Lukacsovich, Jessica Wu, Ricardo Albay, Anna Pensalfini, Stephen Yeung, Elizabeth Head, J Lawrence Marsh, Charles Glabe

**Affiliations:** 1 Department of Molecular Biology and Biochemistry, University of California, Irvine, CA 92697-3900, USA; 2 Department of Pharmacology, University of California San Diego La Jolla, CA 92093-0636, USA; 3 Departments of Developmental and Cell Biology, Pharmacology and the Developmental Biology Center, University of California, Irvine, CA 92697-3900, USA; 4 Sanders-Brown Center on Aging, University of Kentucky, Lexington, KY, 40536-0230, USA; 5 Department of Neurology, University of Texas Medical Branch, Galveston, TX 77555-0144, USA

## Abstract

**Background:**

Age-related neurodegenerative diseases share a number of important pathological features, such as accumulation of misfolded proteins as amyloid oligomers and fibrils. Recent evidence suggests that soluble amyloid oligomers and not the insoluble amyloid fibrils may represent the primary pathological species of protein aggregates.

**Results:**

We have produced several monoclonal antibodies that specifically recognize prefibrillar oligomers and do not recognize amyloid fibrils, monomer or natively folded proteins. Like the polyclonal antisera, the individual monoclonals recognize generic epitopes that do not depend on a specific linear amino acid sequence, but they display distinct preferences for different subsets of prefibrillar oligomers. Immunological analysis of a number of different prefibrillar Aβ oligomer preparations show that structural polymorphisms exist in Aβ prefibrillar oligomers that can be distinguished on the basis of their reactivity with monoclonal antibodies. Western blot analysis demonstrates that the conformers defined by the monoclonal antibodies have distinct size distributions, indicating that oligomer structure varies with size. The different conformational types of Aβ prefibrillar oligomers can serve as they serve as templates for monomer addition, indicating that they seed the conversion of Aβ monomer into more prefibrillar oligomers of the same type.

**Conclusions:**

These results indicate that distinct structural variants or conformers of prefibrillar Aβ oligomers exist that are capable of seeding their own replication. These conformers may be analogous to different strains of prions.

## Background

Many age-related degenerative diseases are characterized by the accumulation of amyloid deposits derived from a variety of proteins. There is conflicting evidence for the role of insoluble fibrillar Aβ deposits in Alzheimer's disease (AD) pathogenesis. The extent of insoluble Aβ plaque accumulation does not correlate well with the severity of dementia in AD [1] and a significant fraction of age matched, non-demented individuals have equivalent amounts of Aβ plaques as demented individuals. In addition, some transgenic animals display cognitive deficits prior to the onset of Aβ plaque accumulation [2,3]. Soluble Aβ levels correlate better with dementia than insoluble, fibrillar deposits [4,5], suggesting that oligomeric forms of Aβ may represent the primary toxic species in AD. Indeed, soluble oligomers have been implicated as primary causative agents in many different degenerative diseases where the accumulation of large fibrillar deposits may be either inert or protective (reviewed in [6,7]).

Conformation dependent, aggregation specific antibodies indicate that there are at least 3 structurally distinct general classes of amyloid oligomers: prefibrillar oligomers, fibrillar oligomers and annular protofibrils. Prefibrillar oligomers (PFOs) are kinetic intermediates that occur at early times of aggregation and are recognized by the polyclonal antibody, A11 [8]. Fibrillar oligomers (FOs) appear to be small pieces of fibril protofilaments that are recognized by the fibril specific polyclonal serum, OC [9,10]. A11 does not recognize amyloid fibrils or monomers while OC does not recognize prefibrillar oligomers or monomers [9]. Annular protofibrils (APFs) are ring shaped, pore-like structures that display an epitope that is specifically recognized by **α**PF antiserum [11]. These three classes of amyloid oligomers appear to be general and common to many different types of amyloid forming proteins and peptides because A11, OC and **α**APF antibodies recognize generic epitopes that are displayed by their respective oligomers when formed by many different peptides of varying amino acid sequences.

Many amyloid fibril structures are known to be parallel, in register β-sheets based on NMR and EPR spectroscopic studies (reviewed in [12,13]), Aβ fibrils are structurally polymorphic and this variation arises from conformational differences in the folding of the parallel, in register sheet [14-16]. These folding polymorphisms are self propagating and give rise to differences in the quaternary structure of the fibrils resulting in the assembly of fibrils containing 2 or 3 protofilaments [14,16,17]. Yeast prions are also parallel, in register structures [18,19] and exhibit remarkable phenotypic variants or strain behavior that may be based on underlying structural variation of the type observed for amyloid fibrils [20]. PFOs appear to be a class of amyloids that is structurally distinct from amyloid fibrils based on the observation that they are recognized in a mutually exclusive manner by the conformation dependent antibodies A11 and OC [9,10]. PFOs also lack strong spin-spin coupling of paramagnetic spin probes that are characteristic of parallel, in-register fibrils [10,21]. Since both of these antibodies recognize generic epitopes that are formed by many different amyloidogenic sequences, these differences are likely to be fundamental differences in the organization of the polypeptides in the sheets, implying that PFOs are not parallel, in-register structures that have been established for amyloid fibrils. FTIR spectroscopy indicates that A11 positive PFOs may be antiparallel beta sheets, implying that they are not intermediates on the pathway to the formation of parallel, in-register fibrils [22]. It is not known whether the PFO structures are polymorphic and display the same types of structural variants that have been reported for amyloid fibrils. Since A11 is polyclonal, it is not clear whether its ability to recognize PFOs from many different types of amyloids is a reflection of the commonality of PFO structure or multiple individual antibodies in the population that recognize different sequence specific PFO epitopes.

We isolated a number of different rabbit monoclonal antibodies (Mabs) specific for prefibrillar oligomers. Here we report that these antibodies recognize generic epitopes that are distributed among several prefibrillar oligomer forming sequences and they display distinct preferences for different protein sequences. In different preparations of Aβ prefibrillar oligomers, the monoclonals recognize distinct subtypes, indicating that structural "strains" of prefibrillar oligomers exist. These strains are capable of propagating by seeding the conversion of Aβ monomer into PFOs of the same type. These results demonstrate that amyloid oligomers are more structurally diverse than was previously known and raise the questions of which of these structural variants is more closely associated with disease pathogenesis and whether the variation contributes to disease heterogeneity.

## Results

### Prefibrillar oligomer specific monoclonals

**Figure 1:**
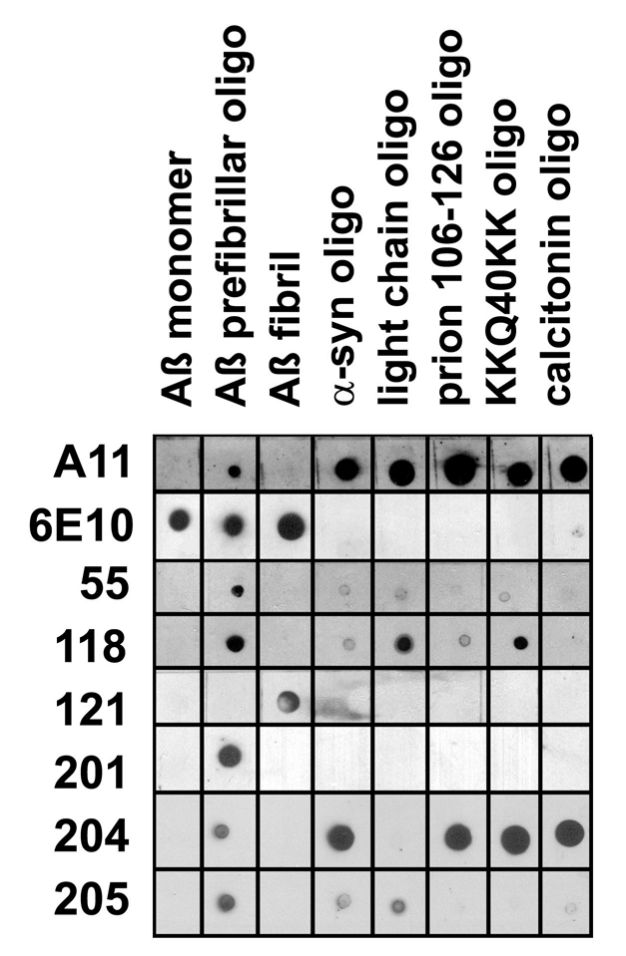
**Dot blot analysis of monoclonal antibody specificity**. Nitrocellulose strips containing 1 ug spots of Aβ40 monomer, PFOs and fibrils and prefibrillar oligomers prepared from α-synuclein, immunoglobulin light chain, prion 106-126 peptide, KK(Q40)KK peptide, and calcitonin were probed with the monoclonal antibodies and with A11 polyclonal and 6E10 control antibodies. A11 stains all prefibrillar oligomers samples, while 6E10 recognizes only samples containing Aβ. The individual monoclonal antibodies only recognize Aβ PFOs and not Aβ monomer or fibril samples, but display distinct preferences for other types of PFOs. Mab204 displays the broadest reactivity, recognizing all PFOs except for light chain, while Mab 201 is the most restrictive reacting with only Aβ PFOs.

We made prefibrillar oligomer specific monoclonal antibodies using the same strategy we employed to make the A11 polyclonal by vaccinating rabbits with Aβ40 covalently coupled to colloidal gold particles via a carboxyl terminal thiol [8]. Rabbits were chosen primarily because we had limited success in making A11-like monoclonals in mice. In 4 independent fusions using 4 different strains of mice, the only antibodies specific for prefibrillar oligomers were IgMs. The rabbit monoclonals we obtained are predominantly IgG and they have the additional advantage that they are the same species of antibodies as the polyclonal A11. We selected 6 different clones based on their differential immunoreactivities on ELISA (data not shown) and dot blot (Figure 1). Four of the 6 clones recognize generic epitopes in several different PFO-forming sequences, including Mabs 55, 118, 204 and 205, which are all IgG1. These antibodies display distinct preferences for different subsets of PFOs, suggesting that the broader range of A11 immunoreactivity is due to the presence of multiple individual antibody specificities. Mab204 appears to have the broadest generic reactivity because it reacts strongly with all PFOs tested except IgG light chain. These results demonstrate that individual clones are capable of recognizing epitopes that are generically distributed among PFOs formed by different sequences and that the different antibodies recognize different epitopes that are differentially displayed on PFOs formed from different sequences. Mab201 appears to be both conformation and sequence specific because it recognizes only Aβ prefibrillar oligomers. This demonstrates that some of the monoclonals are sequence specific and do not recognize generic epitopes. Antibody 121 recognizes Aβ fibrils and not prefibrillar oligomers, similar to OC. Both Mab201 and 121 are IgM class antibodies. Because the hybridoma cells secrete very low amounts of IgM, further work was concentrated on the monoclonal IgGs.

**Figure 2:**
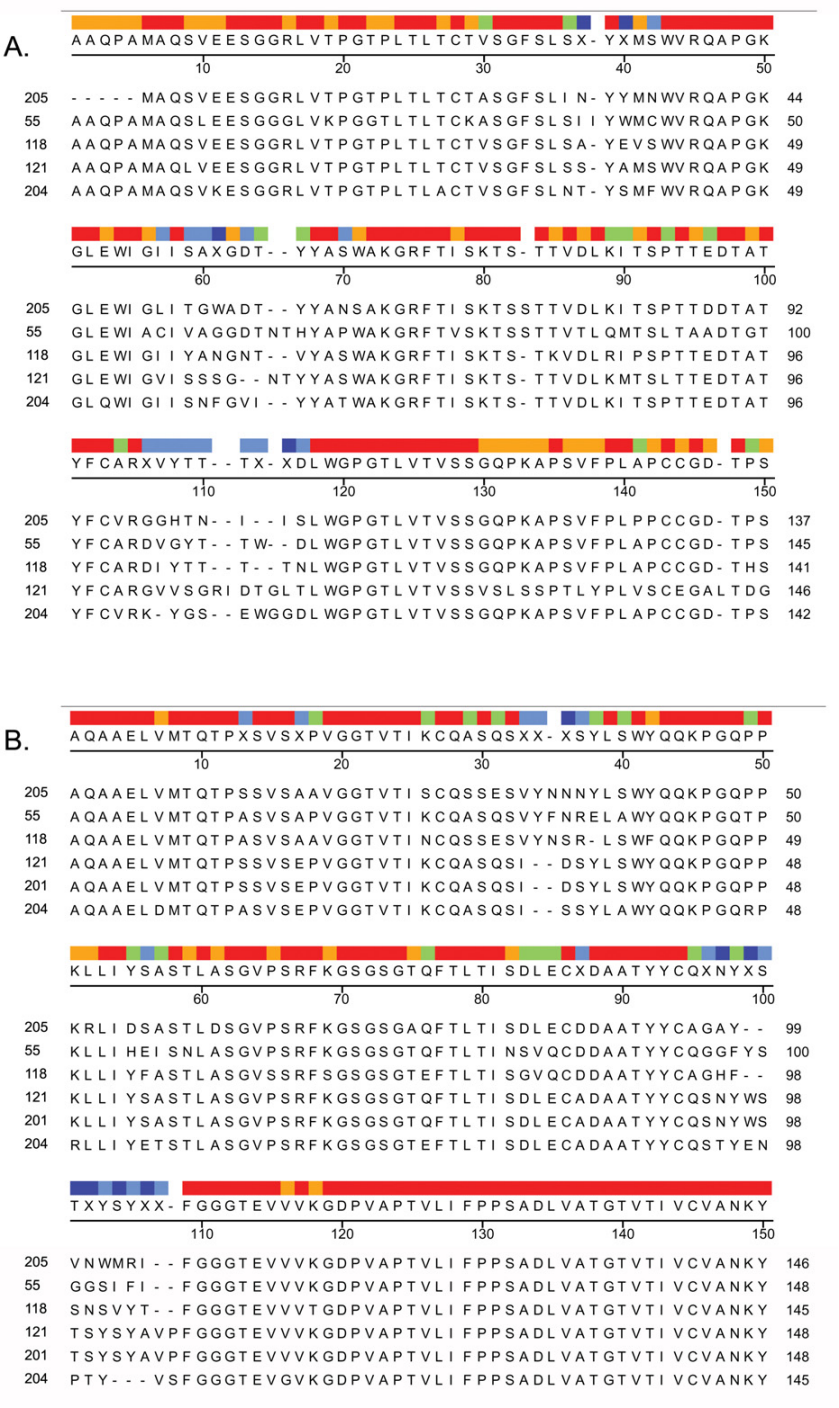
**Sequence comparison of monoclonal antibodies**. Five monoclonal antibody mRNAs were cloned and sequenced. Alignment of heavy chain (A.) and light chain (B.) variable region amino acid sequences by the Clustal V method is shown. Regions of identical sequence are shown in red, while highly variable regions are shown in blue. Regions of increasing similarity are shown in colors of increasing wavelength.

We cloned and sequenced the immunoglobulin heavy and light chains and compared their amino acid sequences (Figure 2A, B). These results indicate that the primary sequences of the 6 monoclonal antibodies are distinct. Mab121 and 201 share a common kappa chain, even though Mab121 is specific for Aβ fibrils, while Mab201 is specific for prefibrillar oligomers. This indicates that the heavy chain is responsible for distinguishing these two conformations of aggregates in 121 and 201. No heavy chain sequence was obtained for 201.

### Monoclonal antibodies identify distinct types of Aβ prefibrillar oligomers

**Figure 3:**
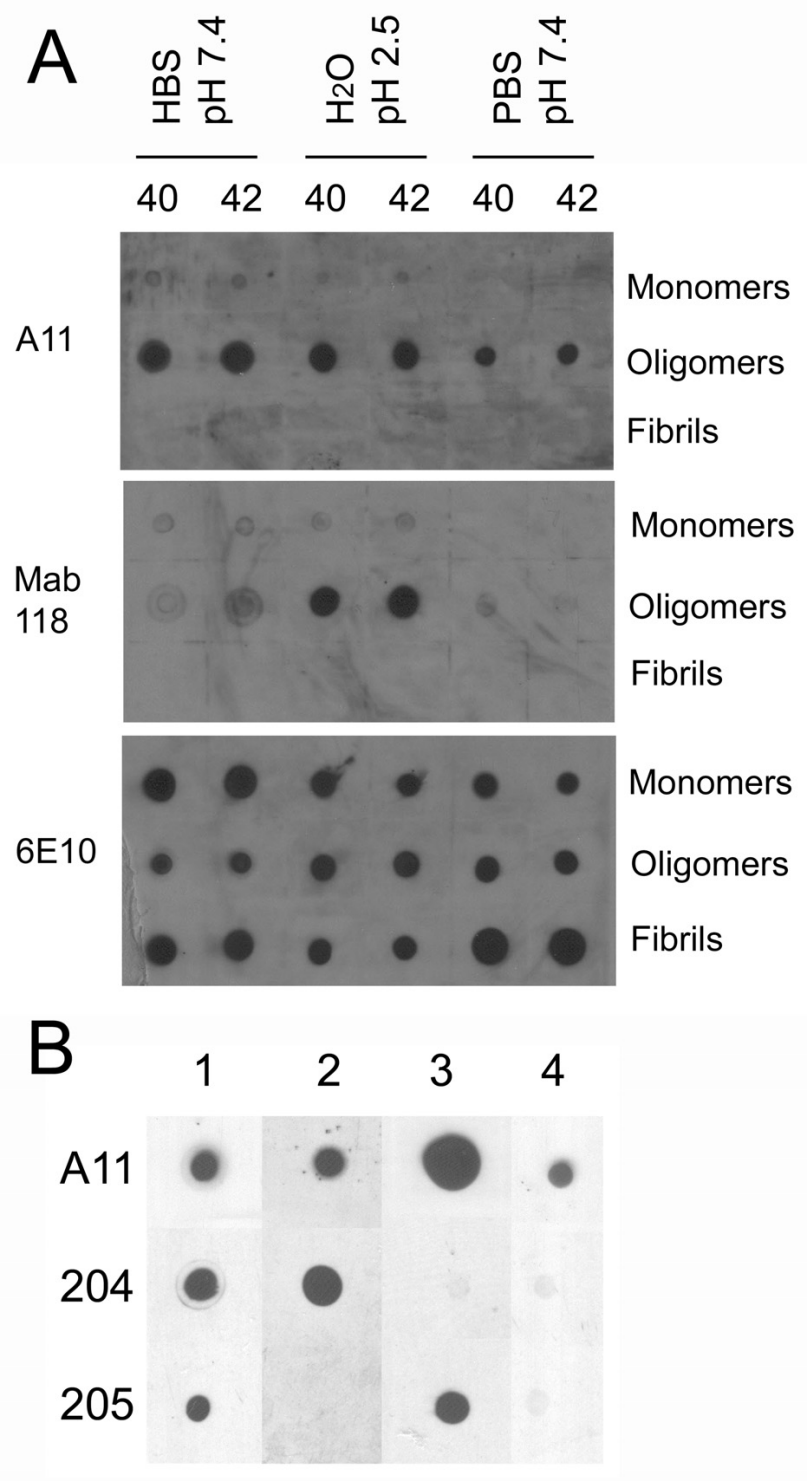
**Monoclonal antibodies distinguish conformational variants of Aβ PFOs**. **A**. Aβ40 (40) and Aβ42 (42) were incubated under three different conditions: HEPES buffered saline pH7.4, water pH 2.5 or PBS pH 7.4. Monomer samples were spotted at time zero, while prefibrillar oligomers and fibrils were spotted at 72 and 240 hours of incubation respectively. All of the oligomer samples stain with A11, but M118 only reacts with the Aβ samples prepared at pH 2.5. **B**. Four different samples of prefibrillar oligomers were prepared in water pH 2.5 and stained with A11, M204 and M205. All samples stain with A11 (top row). Sample 1 stains with both 204 and 205 antibodies while sample 2 stains with only M204, sample 3 stains only with M205 and sample 4 stains with neither monoclonal antibody.

When we examined a large number of A11 positive, Aβ prefibrillar oligomer preparations with the monoclonal IgG antibodies, we observed that some preparations of A11 positive oligomers do not react with some of the monoclonal antibodies, indicating that there are immunologically distinct subclasses of Aβ prefibrillar oligomers (Figure 3). Mab 118 stains both Aβ40 and Aβ42 prefibrillar oligomers prepared at pH 2.5, but does not stain prefibrillar oligomers prepared in Hepes buffered saline (HBS) or phosphate buffered saline (PBS) at pH 7.4 (Figure 3A), suggesting that the epitope for Mab118 is specific to oligomers prepared at pH 2.5. We also examined a number of different prefibrillar oligomer preparations that were all prepared by the same method: dilution from HFIP solution and incubation in water, pH 2.5. These preparations were made from the same lot of peptide using the same protocol on different days over a period of approximately one year. Surprisingly, Mab204 and Mab205 display distinct preferences for the different preparations of Aβ42 oligomers, even though they are all A11 positive (Figure 3B). The reason for the lack of reproducibility in oligomer immunoreactivity in different samples prepared by the same method is not known. These results indicate that there are structural polymorphisms within the class of A11 immunoreactive Aβ prefibrillar oligomers that can be distinguished by the monoclonal antibodies. We have also prepared A11 positive Aβ oligomers that do not react with any of the monoclonal antibodies we have (Figure 3B and data not shown), indicating that there are antibodies in polyclonal A11 that are not represented in the clones we obtained. While the epitope recognized by M118 appears to be pH dependent, it is not yet clear why the immunoreactivities of Mab204 and Mab205 vary because they react differentially with different preparations of Aβ oligomers prepared by the same methods. The variation appears to occur randomly.

### Different types of oligomers have distinct size distributions

**Figure 4:**
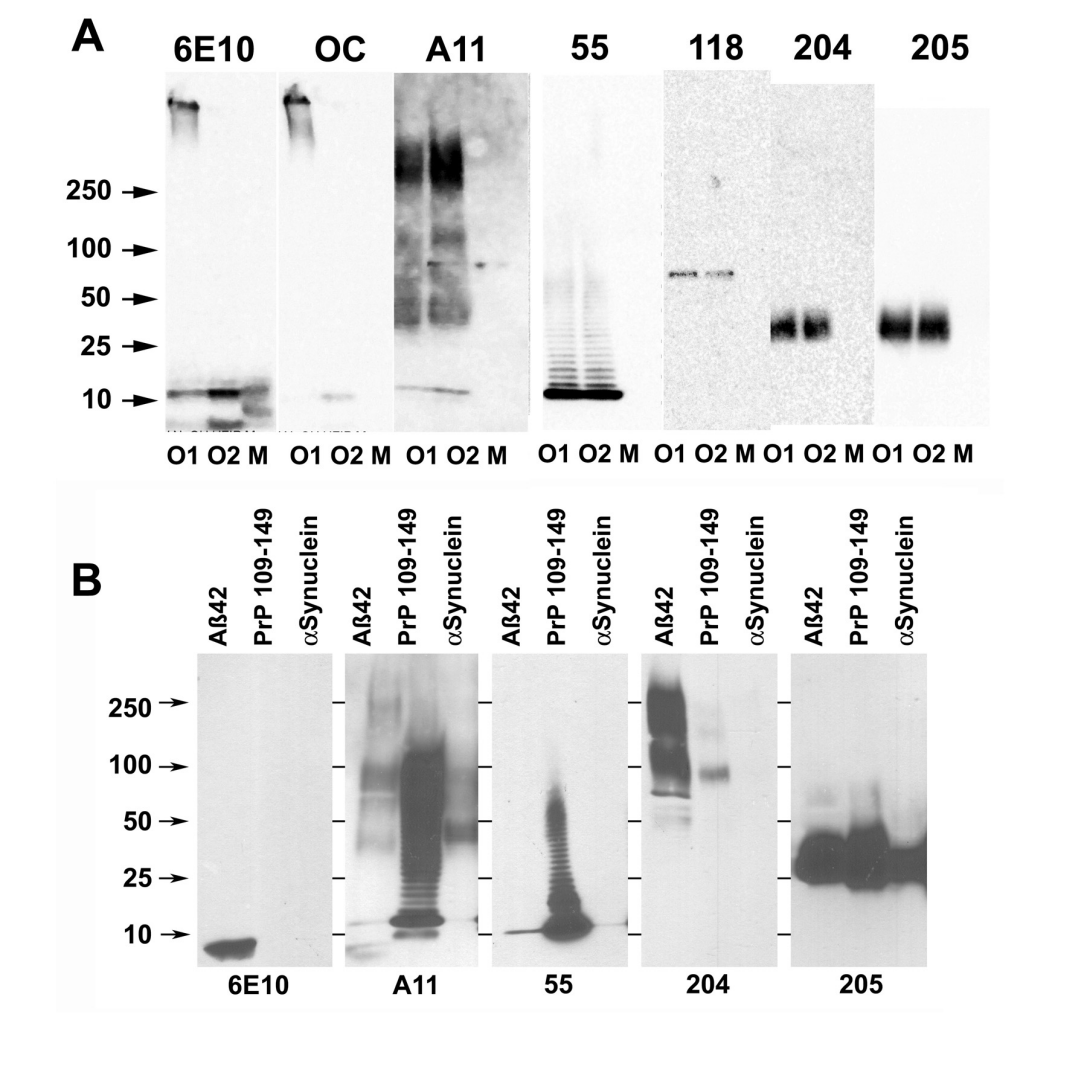
**Western blot analysis of monoclonal antibody PFO specificity**. A. Two different Aβ40 PFOs samples (O1 and O2) and Aβ40 "monomer" (M) were prepared as described in Methods. None of the PFO specific monoclonal antibodies stain Aβ monomer, although the "monomer" sample is recognized by the 6E10 control antibody. Different Mabs stain distinct size distributions of Aβ PFOs. OC polyclonal sera stains high MW bands in oligomer preparation O1 and low MW bands in preparation O2. B. PFO specific monoclonal antibodies recognize soluble oligomers of Aβ42 and other types of amyloids. Soluble oligomers were prepared from Aβ, PrP 109-149 and α-synuclein as described in Methods. Aggregates were analyzed by Western blot with 6E10, A11, and monoclonal antibodies 55, 204, and 205. 6E10 only recognizes Aβ peptide. A11 detects oligomers of different sizes that are formed by Aβ and other types of amyloids. Monoclonal antibodies preferentially recognize different subsets of A11 positive oligomers.

Prefibrillar oligomers were prepared from Aβ40 under two different conditions and analyzed by Western blotting along with freshly prepared monomer. The immunoblots indicate that the monoclonal antibodies recognize distinct size distributions of oligomers (Figure 4A). A11 recognizes a broad range of sizes from a putative dimer of 10 kDa to large oligomers of approximately 240 kDa. The individual monoclonals recognize subsets of the bands stained by A11. Mab55 recognizes a ladder of bands ranging from approximately 12 kDa up to approximately 56 kDa (Figure 4A). The apparent step size for the ladder stained by antibody 55 is approximately 2,250 Da. Since the molecular mass of Aβ40 is 4328 Da, this suggests that the oligomers migrate artifactually fast on SDS gels and that they contain approximately twice the number of peptide chains than their migration on SDS gels would suggest. A similar ladder-like distribution of Aβ oligomers is also observed with A11[10] although it is not apparent on this blot (see below). Mab118 also stains a 56 kDa band but does not detectably stain a dimer band like Mab 55. Mabs204 and 205 stain a broad band between 35 kDa and 45 kDa. The staining of this band by Mab205 is much more intense than that of Mab204. None of the conformation dependent monoclonals or polyclonal antibodies recognize the low molecular weight bands in freshly dissolved Aβ40 preparations (labeled "monomer"), which are recognized by the sequence specific mouse monoclonal antibody 6E10 (Figure 4A). Oligomer preparation 1 contains high molecular weight material migrating at the top of the gel that is stained by the fibril specific polyclonal, OC and by 6E10. This material is commonly observed in fibril preparations, but it is not stained by A11 or any of the A11-like monoclonal antibodies, consistent with the observation that these antibodies do not recognize fibrils on dot blots or ELISA. Additionally, oligomer preparation 2 contains low molecular weight FOs recognized by OC as previously described [9,10]. These results indicate that different sized PFOs display unique epitopes associated with distinct size distributions of oligomers that are a subset of the prefibrillar oligomers stained by the polyclonal A11.

The staining patterns of most of the monoclonal antibodies is the same for the two different preparations of prefibrillar oligomers in Figure 4A, however, Mab204 also stains high molecular weight oligomers in PFOs prepared by dilution of HFIP stock Aβ42 solutions in distilled water and incubated at pH 2.5 (Figure 4B). We also examined the size distribution of PrP 109-149 and α-synuclein PFOs. The size distribution of A11 immunoreactivity for Aβ42 is similar to that of Aβ40 (Figure 4A), while PrP109-149 displayed a ladder-like pattern from approximately 10 kDa to 100 kDa and α-synuclein PFOs ran as a broad band between 36 kDa and 100 kDa. Mab 204 stained high molecular mass bands in Aβ42 PFOs and a 100 kDa band in PrP109-149 PFOs, but did not stain α-synuclein oligomers on Western blots, although Mab204 stained α-synuclein on dot blots (Figure 1). The reason for this differential immunoreactivity is not yet clear, but either the α-synuclein oligomers recognized by Mab204 are sensitive to SDS denaturation or there is conformational variability in these two different α-synuclein oligomer preparations as we have observed for Aβ (Figure 3B). Mab55 stained a 10 kDa Aβ42 band and a ladder-like series of bands in PrP109-149 that are similar to the pattern observed for Aβ40 (Figure 4A). Mab205 stained a broad band between approximately 25-45 kDa in Aβ42, PrP109-149 and α-synuclein. This identical size distribution of the α-synuclein Mab205 immunoreactive species is surprising in view of the fact that the molecular mass of α-synuclein is approximately 3-fold higher than Aβ42 or PrP 109-149. No staining was observed with Mab118 (data not shown). While Mabs55 and 205 stain similar patterns of bands in all samples, the size distribution of Mab204 immunoreactivity varies between Aβ40 and Aβ42 samples.

### Oligomers seed their own replication

**Figure 5:**
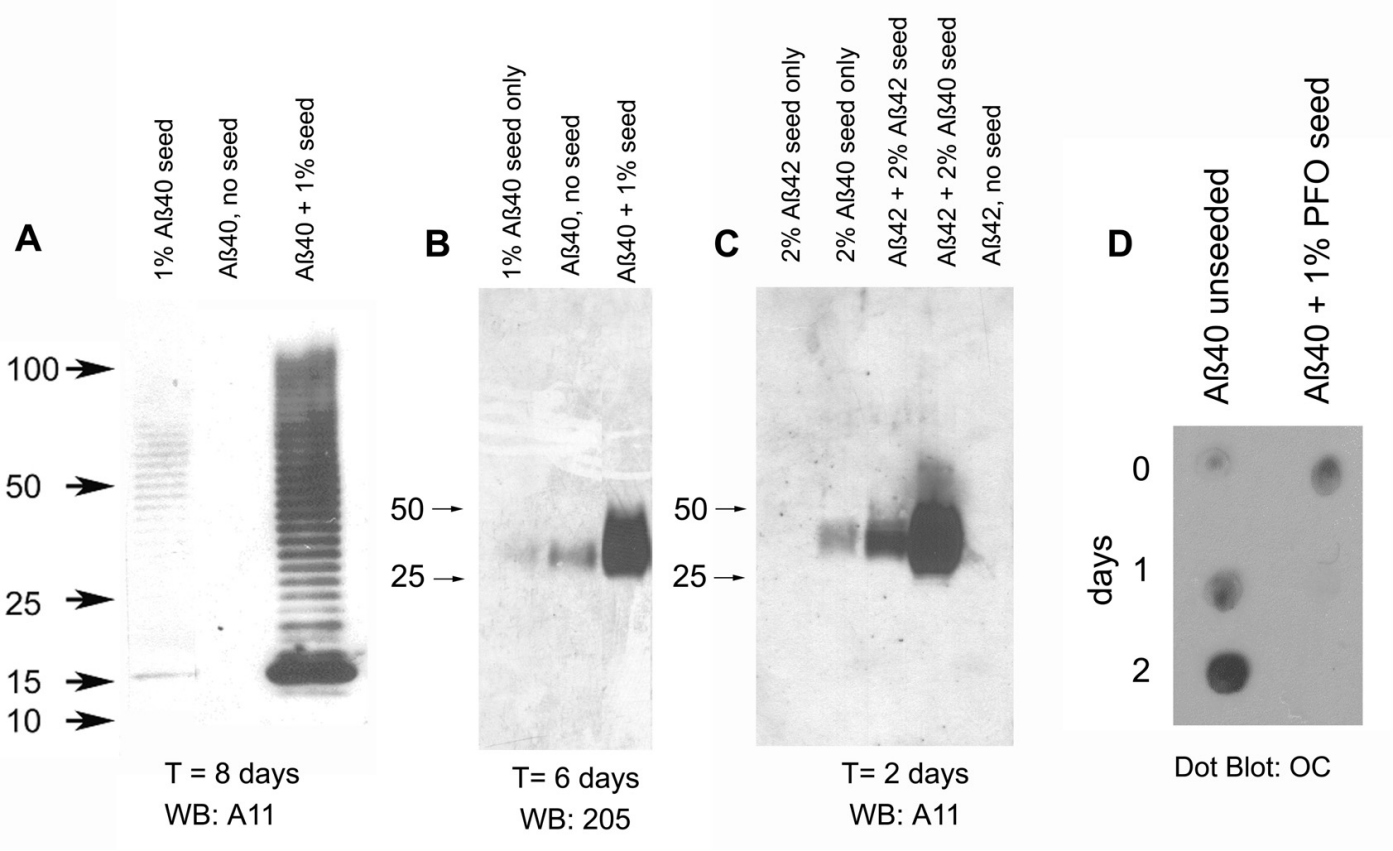
**Prefibrillar oligomers seed prefibrillar oligomer formation from monomer**. A. The presence of a small amount of ladder like 10 - 56 kDa PFO seeds (prepared by dilution of NaOH stock solutions of Aβ40 into10 mM phosphate buffer pH 7.4), accelerates oligomer formation and gives rise to oligomers that stain with A11. No PFO immunoreactivity is observed in the non-seeded samples under these conditions. B. Mab 205 positive 35 - 45 kDa PFOs seed their own formation from Aβ40 monomer. C. Mab 205 35 - 45 kDa Aβ40 and Aβ42 PFOs seed the formation of A11 positive 35 - 45 kDa oligomers from Aβ42 monomers. Only the 35 - 45 kDa band is detected by A11, indicating that other sizes of oligomers are not formed. D. Seeding with PFOs suppresses the formation of fibrillar oligomers. In comparison, FOs spontaneously form in unseeded samples.

Western blotting results with the monoclonal IgGs suggest that there are at least two distinct types of A11 immunoreactive Aβ oligomers with different size distributions that occur randomly in oligomer preparations: A ladder-like series ranging in size from approximately 10 kDa to 56 kDa that is recognized by Mab55 and a broad band between 35 kDa and 45 kDa that is recognized by Mab205. We investigated whether these distinct oligomer types can seed the conversion of Aβ monomer into oligomers of the same immunological type and size distribution. The ladder-like oligomers seed the conversion of monomer into the same type of oligomers as indicated by the acceleration of the formation of A11 positive oligomers compared to unseeded monomer controls (Figure 5A). The rate of formation is slow compared to fibril and fibrillar oligomer seeded polymerization [10], reaching a maximum in approximately 8 days. However, no A11 oligomers are observed under these conditions in the unseeded samples. Similar results were obtained when Aβ40 monomer was seeded with 1% of a preparation of Mab205 positive Aβ40 prefibrillar oligomers that run as a broad band between 35 - 45 kDa (Figure 5B). The presence of seeds promotes the formation of Mab205 positive oligomers compared to monomer samples incubated in the absence of seeds. Aβ40 and Aβ42 35 - 45 kDa oligomers also seeded the oligomerization of Aβ42 to form A11 positive oligomers that run as a broad band between 35-45 kDa (Figure 5C). The absence of staining of other size bands by A11 indicates that the seeding results in the template specific assembly of a homogeneous population of 35-45 kDa oligomers.

Fibrils and fibrillar oligomers spontaneously form in solutions of Aβ40 monomer in H_2_O at pH 7.4 [10]. These fibrillar aggregates are recognized by OC polyclonal antiserum, but not by A11[9]. We examined whether seeding these solutions with PFOs can compete for fibril and fibrillar oligomer formation (Figure 5D). The addition of 1% PFO seeds completely suppresses the development of OC positive fibrils and fibrillar oligomers over two days of incubation. Western blot analysis of the samples showed that A11 positive PFOs developed in this sample rather than OC positive fibrils and FOs (data not shown). These results indicate that the presence of PFOs alters the aggregation pathway to favor the formation of PFOs rather than FOs that would normally form in the absence of PFO seeds.

### Oligomer specific monoclonal antibodies do not stain plaques

**Figure 6:**
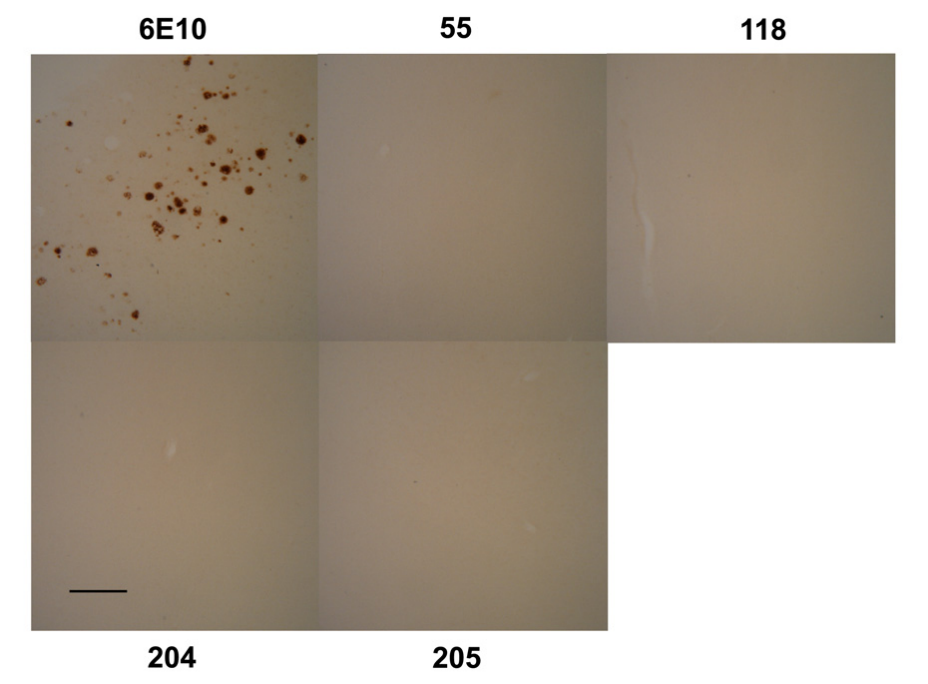
**A11-like monoclonal antibodies do not stain amyloid plaques in human AD brain**. Sections of frontal cortex (Broadman's area 11) were stained with Mabs 55, 18, 204 and 205 and 6E10 as a control. The bar indicates 200 μm.

We also investigated whether the monoclonal IgGs can detect the accumulation of PFOs in human AD brain and Tg2576 and 3xTg-AD transgenic mouse brains. No specific staining of plaque deposits was observed by immunohistochemistry in human AD brain (Figure 6) or transgenic mouse brain (data not shown), indicating that the Mabs do not stain plaques, consistent with their lack of reactivity with amyloid fibrils. It is not yet clear whether the oligomers recognized by these antibodies are absent from human AD or Tg mouse brain tissue. The antibodies exhibit low background reactivity on tissue, indicating that they do not detectably cross react with normal proteins. Further experiments with higher resolution and sensitivity, such as immuno electron microscopy and immunoprecipitation will be necessary to determine whether these oligomers are detectable in vivo.

## Discussion

The prefibrillar amyloid oligomer specific monoclonal antibodies that we have cloned display the canonical features of A11, the corresponding polyclonal antiserum from which they are derived. They recognize epitopes that are specifically associated with prefibrillar oligomers and do not recognize fibrils, fibrillar oligomers, monomer or natively folded proteins. Like A11, the monoclonal IgGs recognize generic epitopes that are displayed on PFOs from several different amyloidogenic sequences, but one of the IgM antibodies, Mab201, recognizes only Aβ PFOs, indicating that some of the epitopes are both conformation and sequence specific. Unlike A11, which displays no obvious sequence preference, the individual monoclonals display distinct preferences for different types of oligomers. All of the monoclonal IgGs react strongly with Aβ PFOs because this preparation was used in the primary screening protocol to select the immunopositive hybridomas. Mab55 appears to be the most selective, because it only reacts weakly with other types of PFOs on dot blots. Mab204 appears to have the broadest specificity as it recognizes all types of PFOs strongly except light chain.

The distinct PFO preferences displayed by the monoclonal IgGs would appear to indicate that the antibodies prefer PFOs formed by certain sequences and not others, but further analysis indicates that conformational polymorphism within a sequence is another plausible explanation. The surprising finding is that the same type of immunological variation is observed in different preparations of Aβ. This indicates that the different epitopes can be displayed or not by the same sequence depending on the conformation of the peptide and suggests that the Aβ sequence can adopt a number of distinct conformations that may be analogous to the structural polymorphisms in different strains of the yeast prion Sup35 [23]. The variation in detection of Aβ PFOs by Mabs204 and 205 indicates that there are at least three distinct types of PFOs: Mab204+, Mab205+ and a type that is negative for both Mabs that is recognized by A11. The fact that none of the Mabs reacted with this latter preparation of Aβ PFOs suggests that some of the antibodies represented in A11 have yet to be cloned.

One of the key properties of yeast prions is the ability to replicate and propagate in a template specific fashion [24], so we investigated the ability of Aβ PFOs to seed the conversion of Aβ monomer in a template specific fashion. We found that both the ladder like Mab55 positive PFOs and the 36 kDa Mab205 positive PFOs are capable of accelerating the conversion of Aβ monomer into the same type of PFOs used to seed the reaction, indicating that PFOs also seed in a template specific fashion. Fibrils and fibrillar oligomers are also capable of seeding the conversion of monomer in a template specific fashion [10,14]. This indicates that several distinct types of Aβ oligomers exist that have structurally distinct lattices that can facilitate the addition of monomers by intermolecular hydrogen bonding to the ends of the beta sheets. The newly added Aβ peptide adopts the same folded conformation as the peptides serving as the template beneath it extending the intermolecularly hydrogen bonded β sheet lattice. The stability of the lattice appears to determine the rate at which the Aβ oligomers replicate as it does for yeast prions [10]. Aβ40 fibrillar oligomers replicate maximally in approximately 2 hours [10], while Aβ40 PFOs take several days to seed the conversion of monomer into PFOs.

The observation of structurally distinct strains of Aβ prefibrillar oligomers that display different size distributions on Western blots may provide some insight into the structural organization of this class of oligomers. The fact that none of the PFO specific Mabs recognize fibrillar structures that are known to be parallel β strands in exact register suggests that PFOs are not parallel beta sheets. The generic epitopes associated with parallel β sheet fibrils are recognized by OC polyclonal antibodies that are specific for fibrils and fibrillar oligomers and do not recognize PFOs [9,10]. A molten globule like intermediate has been proposed for amyloid aggregation [25-27]. In computer simulations of Aβ peptide aggregation, monomers were observed to rapidly coalesce into a "molten oligomer" ensemble of disordered peptides held together by predominantly hydrophobic interactions and at later times evolve into intermolecularly hydrogen bonded oligomers [28]. It seems unlikely that an ensemble of disordered molten structures would display unique epitopes that the antibodies could discriminate, so this class of oligomer structure does not seem to have the requisite structural diversity to explain all of the types of oligomers observed here. However, the disordered molten oligomer could constitute one of the oligomer subtypes that are not recognized by the conformation dependent antibodies. It has also been suggested that A11 positive PFOs are anti-parallel β sheets, based on FTIR spectroscopy [22]. This model provides a facile explanation for the difference between fibrils and fibrillar oligomers, which are parallel beta sheets and PFOs, but it raises the question of the structural basis for the conformational polymorphisms and the molecular nature of the generic epitopes. If PFOs are ordered, antiparallel β sheets, then the strands would alternate, giving rise to a linear pattern of alternating amino acid side chains displayed on the surfaces of the sheet. These tracts of alternating side chains could constitute the generic epitopes for the A11 like Mabs, similar to the fashion in which the homogeneous amino side chain tracts may constitute the generic fibril epitopes on parallel in register β sheets. Depending on the folding of the sheet, these epitopes may be either hidden or exposed on the surface of the sheet, which could explain the diverse pattern of immunological recognition of Aβ PFOs by the Mabs.

Alzheimer's disease is known to be clinically and pathologically heterogeneous [29]. Moreover, the role of amyloid in disease is inconsistent with some cognitively normal individuals having large amounts of amyloid plaques, suggesting that not all amyloid is pathologically significant [30]. The findings that amyloid fibrils [14,16] and prefibrillar oligomers display structural heterogeneity suggests that these polymorphisms may underlie the disease heterogeneity and predict that the different structural variants may have distinct toxic activities and pathological significance.

## Methods

### Antibody production

Rabbit monoclonal antibodies were produced under a contract with Epitomics, Incorporated, Burlingame, CA. New Zealand white rabbits were immunized subcutaneously with Aβ40-colloidal gold oligomer mimics at a 0.25 mg Aβ dose bit and boosted seven times with the same amount at 3 weeks intervals. The titer and specificity of the immune response was determined by ELISA and dot blot analysis as previously described using serum collected after the third and sixth boosts [8]. The ELISA plates and dot blot strips were coated with 50 ng of 5 different samples: Aβ monomer, Aβ PFOs, Aβ fibrils, α-synuclein PFOs and islet amyloid polypeptide (IAPP) PFOs. Rabbits demonstrating a serum titer ≥ 0.3 OD at dilution of 1:64,000 were used as a source of splenocytes for fusion. A11 and 6E10 antibodies were used as positive controls and preimmune serum was used as negative control.

### Hybridoma screening

96 hybridoma pools out of a total of 1920 were selected that were positive for Aβ42 PFOs by ELISA using plates coated with 50 nm of Aβ42 PFO. Of this set of 96 pools, 36 pools displayed aggregation state specificity for Aβ42 PFOs were selected for further analysis by ELISA using plates coated with Aβ monomer, Aβ PFO, Aβ fibrils, α-synuclein PFO and IAPP PFO, and dot blotting against Aβ monomer, PFOs, Aβ fibrils and alpha synuclein, light chain, PrP 106-126, KKQ40KK and calcitonin oligomers. Based on these analyses, 6 clones displaying unique immunoreactivities were selected for cloning: 55, 118, 121, 201, 204 and 205.

### Preparation of Aβ Oligomers

Lyophilized peptide (Aβ 40 or 42) was resuspended in 50% acetonitrile/water mixture and relyophilized. Soluble oligomers were prepared by dissolving 0.3 mg of the peptide in 250 μl hexafluoroisopropanol (HFIP) and incubating for 10-20 min at room temperature. The resulting solution was diluted to 70 μM in H_2_O containing 0.02% NaN_3 _in a siliconized Eppendorf tube as previously described [8]. In some experiments, the HFIP stock solution was diluted in Hepes buffered saline, pH 7.4 (HBS) or phosphate buffered saline pH 7.4 (PBS) containing 0.02% NaN_3_. The samples were then stirred at 500 RPM using a Teflon-coated micro stir bar for at room temperature. Aliquots were taken at 0-240 hr intervals for testing with anti-oligomer antibody A11 [8]. Alternatively Aβ40 oligomers were prepared by dissolving the lyophilized peptide (0.3 mg) at a concentration of 2.5 mM in 100 mM NaOH. The oligomerization reaction was initiated by diluting the stock solution to 70 μM in 10 mM phosphate buffer, pH 7.4, 0.02% NaN_3_. The monomer was prepared by dissolving the Aβ 40 (0.1 mg) in 30 μl of 10%SDS, heating at 90°C for 5 minutes and diluted to 45 μM in H_2_O. PrP 109-149, and α-synuclein were solubilized in HFIP (hexafluoro-2-propanol) at a concentration of 420 uM for 25 minutes at room temperature. Then the peptide solution was diluted into ddH2O, pH 2.5, 0.02% NaN_3 _at 70 μM and stirred at 500 rpm. with a Teflon coated micro stir bar for 2 days. Caps with three 18 gauge needle holes were used to allow slow evaporation of HFIP.

### Western and dot blotting

Samples containing 4 μg of Aβ42, PrP 109-149, and α-synuclein were dissolved in SDS treatment buffer, boiled for 5 min, and electrophoresed on 10-20% Tris-HCl (Bio-Rad) gels. Proteins were electrophoretically transferred onto nitrocellulose membranes and developed with 6E10 (0.1 ug/ml), A11 (1.05 ug/ml), and monoclonal antibodies 48 (1.15 ug/ml), 55 (0.8 ug/ml), 204 (1.05 ug/ml), and 205(0.2 ug/ml). Dot blots and Western blots were performed as previously described [10]. For Western blots, samples containing 4 μg of Aβ40 were dissolved in SDS treatment buffer, boiled for 5 min, and electrophoresed on 4-20% Tris-HCl (Bio-Rad) gels. Proteins were electrophoretically transferred onto nitrocellulose membranes and developed with conformation-specific antibodies. For dot blots, 0.36 ug of protein was spotted. The concentration of primary antibodies were 6E10 (0.1 μg/ml/ml), OC (0.2 μg/ml), A11 (1.05 μg/ml), 48(1.15 μg/ml), 55(0.8 μg/ml), 118 (0.2 μg/ml), 204 (1.05 μg/ml) and 205 (0.2 μg/ml). The membranes were then incubated with anti-rabbit IgG conjugated with horseradish peroxidase (1:10,000, Jackson) at room temperature for 1 h. The blots were developed with Super signal West pico chemiluminescence kit from Thermo Scientific.

### Oligomer seeding

Aβ40 peptide (0.05 mg) was dissolved in sodium hydroxide (0.1 M, 12 μl) and incubated for 30 min. The solution was then diluted with sodium phosphate buffer (10 mM, pH 8.0, 500 μl), and then seeded with previously prepared A11-positive oligomers (10 μl, 0.2 mg/ml, 4% by weight). The reaction mixture was incubated at 24°C for 2-10 days and assayed for antibody reactivity with Western and dot blots. The diluted Aβ40 solution in the absence of added oligomers was used as a control.

### Immunohistochemistry

Fixed brain tissues (prefrontal cortex) from AD patients were sectioned (50 μm) with a vibratome. Coronal sections were collected in PBS (containing 0.02% sodium azide) and stored at 4°C prior to staining. To stain for Aβ plaques, sections were immersed in 70% formic acid for 5 min. Endogenous peroxidase in tissue was blocked by treating with 3% H_2_O_2 _in PBS for 10 min at room temperature. Nonspecific background staining was blocked by 1 h incubation in 2% BSA, 0.3% Triton X-100 (TX) at room temperature. Tissues were incubated with primary antibodies 6E10 (0.2 μg/ml), 55(3.2 μg/ml), 118(1.6 μg/ml), 204 (4.8 μg/ml) and 205 (4 μg/ml) for 48 hrs at 4°C, rinsed 3 times with PBS,0.1% TX, followed by biotinylated secondary antibodies (anti-mouse for 6E10 and anti-rabbit for rabbit antibodies), detection with an ABC peroxidase kit, and visualization with a 3,3'-diaminobenzidine (DAB) substrate kit (Vector, Burlingame, CA).

## Competing interests

The authors declare that they have no competing interests.

## Authors' contributions

RK prepared the antigens and oligomer samples and did the initial screening and characterization of the monoclonal antibodies. SY prepared antibodies and characterized them. EH, and RA did the immunohistochemistry on transgenic mouse and human AD tissue. JW, LB, AP, IC and SR prepared oligomer samples and conducted the dot blot and Western blot analysis of the samples. LM and TL cloned and sequenced the heavy and light chain cDNAs. CG participated in concept, design, data analysis and manuscript preparation. All authors read and approved the final manuscript.
